# Supplementary material for: The LEPR Gene Is Associated with Reproductive Seasonality Traits in Rasa Aragonesa Sheep
Source: Animals (Basel). 2020 Dec 21;10(12):2448. doi: 10.3390/ani10122448 (PMC7766475; doi:10.3390/ani10122448)
Supplement: Supplementary file 1 [file animals-10-02448-s001.zip › Table S1.docx]

**Table S1.** Genotypic and allelic frequencies of the identified SNPs

| **Location** | **Alias** | **dbSNPs** | **Genotype** | **Genotype frequencies** | **Allele** | **Allele frequencies** |
| --- | --- | --- | --- | --- | --- | --- |
| Exon 4 | snp_ex4 | rs411478947 | AA | 0.004 | G | 0.935 |
|  |  |  | AG | 0.123 | A | 0.065 |
|  |  |  | GG | 0.873 |  |  |
| Exon 7 | snp_ex7 | rs596133197 | CC | 0.954 | C | 0.977 |
|  |  |  | CT | 0.046 | T | 0.023 |
| Exon 8 | snp_ex8 | rs403578195 | CC | 0.878 | C | 0.939 |
|  |  |  | GC | 0.122 | G | 0.061 |
| Exon 20 | snp_ex20_1 | rs412929474 | AA | 0.026 | G | 0.893 |
|  |  |  | AG | 0.163 | A | 0.107 |
|  |  |  | GG | 0.811 |  |  |
|  | snp_ex20_2 | rs428867159 | CC | 0.686 | C | 0.822 |
|  |  |  | CT | 0.271 | T | 0.178 |
|  |  |  | TT | 0.043 |  |  |
|  | snp_ex20_3 | rs405459906 | AA | 0.621 | A | 0.793 |
|  |  |  | AG | 0.344 | G | 0.207 |
|  |  |  | GG | 0.035 |  |  |
