# Supplementary material for: The LEPR Gene Is Associated with Reproductive Seasonality Traits in Rasa Aragonesa Sheep
Source: Animals (Basel). 2020 Dec 21;10(12):2448. doi: 10.3390/ani10122448 (PMC7766475; doi:10.3390/ani10122448)
Supplement: Supplementary file 1 [file animals-10-02448-s001.zip › Table S2.docx]

**Table S2.** Type III test for the body condition (BC), live weight (LW), age (A), SNP, and SNP x age effects for the *LEPR* polymorphisms using the seasonality phenotype data from Rasa Aragonesa ewes. The least square means (LSMs) and standard errors of the *LEPR* polymorphisms in the seasonality phenotype data in Rasa Aragonesa ewes are also shown.

| **SNP** |  |  | **P value** | | | | | |  |  | **LSMs SNP** | | |  | **A**^1^ | **LSMs SNP x A** | | |
| --- | --- | --- | --- | --- | --- | --- | --- | --- | --- | --- | --- | --- | --- | --- | --- | --- | --- | --- |
|  |  |  | **Phenotype** | **BC** | **LW** | **A** | **SNP** | **SNP x A** |  |  |  |  |  |  |  |  |  |  |
|  |  |  |  |  |  |  |  |  |  |  |  |  |  |  |  |  |  |  |
| snp_ex4 |  |  |  |  |  |  |  |  |  |  | AA | AG | GG |  |  | AA | AG | GG |
|  |  |  | TDA | 0.435 | 0.021 | 0.072 | 0.688 | 0.996 |  |  | - | 72.6±9.71 | 68.3±3.29 |  | M | 93.1±44.28 | 65±9.65 | 60.7±4.42 |
|  |  |  |  |  |  |  |  |  |  |  |  |  |  |  | Y | - | 80.2±17.33 | 76±6.28 |
|  |  |  | P4CM | 0.126 | 0.119 | 0.088 | 0.361 | 0.596 |  |  | - | 0.78±0.03 | 0.82±0.01 |  | M | 0.74±0.16 | 0.79±0.03 | 0.85±0.01 |
|  |  |  |  |  |  |  |  |  |  |  |  |  |  |  | Y | - | 0.77±0.06 | 0.79±0.02 |
|  |  |  | OCM | 0.117 | 0.052 | 0.364 | 0.836 | 0.856 |  |  | - | 0.53±0.04 | 0.51±0.01 |  | M | 0.48±0.20 | 0.56±0.04 | 0.53±0.02 |
|  |  |  |  |  |  |  |  |  |  |  |  |  |  |  | Y | - | 0.51±0.07 | 0.50±0.02 |
| snp_ex7 |  |  |  |  |  |  |  |  |  |  | CC | CT |  |  |  | CC | CT |  |
|  |  |  | TDA | 0.398 | 0.015 | 0.075 | 0.461 | - |  |  | 68.2±3.14 | - | - |  | M | 60.7±4.24 | 70.9±13.39 | - |
|  |  |  |  |  |  |  |  |  |  |  |  |  |  |  | Y | 75.7±6.11 | - | - |
|  |  |  | P4CM | 0.097 | 0.114 | 0.084 | 0.179 | - |  |  | 0.81±0.01 | - | - |  | M | 0.84±0.01 | 0.77±0.05 | - |
|  |  |  |  |  |  |  |  |  |  |  |  |  |  |  | Y | 0.79±0.02 | - | - |
|  |  |  | OCM | 0.118 | 0.052 | 0.319 | 0.740 | - |  |  | 0.52±0.01 | - | - |  | M | 0.54±0.01 | 0.52±0.06 | - |
|  |  |  |  |  |  |  |  |  |  |  |  |  |  |  | Y | 0.50±0.02 | - | - |
| snp_ex8 |  |  |  |  |  |  |  |  |  |  | CC | GC |  |  |  | CC | GC |  |
|  |  |  | TDA | 0.361 | 0.014 | 0.120 | 0.072 | 0.643 |  |  | 66.3±3.33 | 82.4±8.21 | - |  | M | 60.3±4.26 | 72.4±11.51 | - |
|  |  |  |  |  |  |  |  |  |  |  |  |  |  |  | Y | 72.2±6.48 | 92.4±12.26 | - |
|  |  |  | P4CM | 0.103 | 0.099 | 0.141 | 0.379 | 0.642 |  |  | 0.82±0.01 | 0.79±0.03 | - |  | M | 0.84±0.01 | 0.82±0.04 | - |
|  |  |  |  |  |  |  |  |  |  |  |  |  |  |  | Y | 0.80±0.02 | 0.75±0.04 | - |
|  |  |  | OCM | 0.081 | 0.045 | 0.451 | 0.003 | 0.075 |  |  | 0.54±0.01**a** | 0.42±0.03**b** | - |  | M | 0.54±0.01 | 0.49±0.05 | - |
|  |  |  |  |  |  |  |  |  |  |  |  |  |  |  | Y | 0.53±0.02 | 0.34±0.05 | - |
| snp_ex20_1 |  |  |  |  |  |  |  |  |  |  | AA | AG | GG |  |  | AA | AG | GG |
|  |  |  | TDA | 0.291 | 0.039 | 0.091 | 0.525 | 0.0004 |  |  | 84.8±18.76 | 63.5±7.29 | 69.9±3.33 |  | M | 89±21.52 | 81.4±8.87 | 56.5±4.51 |
|  |  |  |  |  |  |  |  |  |  |  |  |  |  |  | Y | 80.6±31.09**ab** | 45.6±11.98**a** | 83.3±6.41**b** |
|  |  |  | P4CM | 0.064 | 0.217 | 0.123 | 0.558 | 0.002 |  |  | 0.74±0.07 | 0.83±0.02 | 0.81±0.01 |  | M | 0.77±0.08 | 0.77±0.03 | 0.85±0.01 |
|  |  |  |  |  |  |  |  |  |  |  |  |  |  |  | Y | 0.71±0.11 | 0.89±0.04 | 0.76±0.02 |
|  |  |  | OCM | 0.172 | 0.047 | 0.366 | 0.439 | 0.012 |  |  | 0.47±0.08 | 0.56±0.03 | 0.51±0.01 |  | M | 0.39±0.09 | 0.50±0.04 | 0.55±0.02 |
|  |  |  |  |  |  |  |  |  |  |  |  |  |  |  | Y | 0.56±0.14 | 0.63±0.05 | 0.47±0.02 |
| snp_ex20_2 |  |  |  |  |  |  |  |  |  |  | CC | CT | TT |  |  | CC | CT | TT |
|  |  |  | TDA | 0.348 | 0.028 | 0.073 | 0.106 | 0.020 |  |  | 67.9±3.66 | 67.5±5.71 | 92.2±14.12 |  | M | 54.6±4.75 | 70.5±7.05 | 103.4±17.75 |
|  |  |  |  |  |  |  |  |  |  |  |  |  |  |  | Y | 81.2±6.87 | 64.4±9.70 | 80.9±22.17 |
|  |  |  | P4CM | 0.078 | 0.176 | 0.101 | 0.160 | 0.061 |  |  | 0.81±0.01 | 0.82±0.02 | 0.72±0.05 |  | M | 0.86±0.01 | 0.81±0.02 | 0.70±0.06 |
|  |  |  |  |  |  |  |  |  |  |  |  |  |  |  | Y | 0.77±0.02 | 0.84±0.03 | 0.75±0.08 |
|  |  |  | OCM | 0.190 | 0.050 | 0.304 | 0.104 | 0.124 |  |  | 0.53±0.01 | 0.51±0.02 | 0.42±0.06 |  | M | 0.57±0.02 | 0.49±0.03 | 0.38±0.08 |
|  |  |  |  |  |  |  |  |  |  |  |  |  |  |  | Y | 0.49±0.03 | 0.53±0.04 | 0.45±0.10 |
| snp_ex20_3 |  |  |  |  |  |  |  |  |  |  | AA | AG | GG |  |  | AA | AG | GG |
|  |  |  | TDA | 0.420 | 0.034 | 0.071 | 0.076 | 0.166 |  |  | 67±3.92 | 67.7±5.36 | 99±16.14 |  | M | 54.4±5.26 | 66.5±6.38 | 103±19.74 |
|  |  |  |  |  |  |  |  |  |  |  |  |  |  |  | Y | 79.6±7.25 | 68.8±9.33 | 95.1±25.74 |
|  |  |  | P4CM | 0.142 | 0.153 | 0.117 | 0.148 | 0.172 |  |  | 0.81±0.01 | 0.83±0.02 | 0.70±0.06 |  | M | 0.86±0.01 | 0.82±0.02 | 0.72±0.07 |
|  |  |  |  |  |  |  |  |  |  |  |  |  |  |  | Y | 0.77±0.02 | 0.83±0.03 | 0.69±0.09 |
|  |  |  | OCM | 0.195 | 0.062 | 0.261 | 0.019 | 0.283 |  |  | 0.54±0.01 | 0.50±0.02 | 0.38±0.07 |  | M | 0.58±0.02 | 0.49±0.02 | 0.39±0.08 |
|  |  |  |  |  |  |  |  |  |  |  |  |  |  |  | Y | 0.50±0.03 | 0.50±0.04 | 0.37±0.11 |

^1^M=mature; Y=young
