# Supplementary material for: The LEPR Gene Is Associated with Reproductive Seasonality Traits in Rasa Aragonesa Sheep
Source: Animals (Basel). 2020 Dec 21;10(12):2448. doi: 10.3390/ani10122448 (PMC7766475; doi:10.3390/ani10122448)
Supplement: Supplementary file 1 [file animals-10-02448-s001.zip › Table S3.docx]

**Table S3**: Haplotypes combination and frequency for block 0 (rs411478947-rs596133197-rs403578195-rs412929474-rs428867159-rs405459906), block 1 (rs411478947-rs596133197-rs403578195) and 2 (rs412929474-rs428867159-rs405459906). Only haplotypes with a frequency higher than 1% are shown.

|  | **Haplotype** | **Combination** | **Frequency** |
| --- | --- | --- | --- |
| Block 0 | h1 | GCCATG | 0.089 |
|  | h2 | GCCGTG | 0.072 |
|  | h3 | GCCGCG | 0.025 |
|  | h4 | GC**G**GCA | 0.037 |
|  | h5 | A**T**CGCA | 0.017 |
|  | h6 | ACCGCA | 0.039 |
|  | h7 | GCCGCA | 0.688 |
|  | h8 | GC**G**ATG | 0.012 |
| Block 1 | h1 | GCG | 0.053 |
|  | h2 | ATC | 0.019 |
|  | h3 | ACC | 0.045 |
|  | h4 | GCC | 0.878 |
| Block 2 | h1 | ATG | 0.103 |
|  | h2 | GTG | 0.073 |
|  | h3 | GCG | 0.033 |
|  | h4 | GCA | 0.786 |
