# Supplementary material for: The LEPR Gene Is Associated with Reproductive Seasonality Traits in Rasa Aragonesa Sheep
Source: Animals (Basel). 2020 Dec 21;10(12):2448. doi: 10.3390/ani10122448 (PMC7766475; doi:10.3390/ani10122448)
Supplement: Supplementary file 1 [file animals-10-02448-s001.zip › Table S4.docx]

**Table S4.** Type III test for the body condition (BC), live weight (LW), age (A), haplotype (H), and haplotype x age (H x A) effects for the Block 0 haplotype using the seasonality phenotype data from Rasa Aragonesa ewes. The least square means (LSMs) and standard errors of the *LEPR* polymorphisms in the seasonality phenotype data in Rasa Aragonesa ewes are also shown.

|  |  |  | **P value** | | | | | |  | **LSMs H**^2^ | | |  | **A**^3^ | **LSMs H x A**^2^ | | | | | |  |
| --- | --- | --- | --- | --- | --- | --- | --- | --- | --- | --- | --- | --- | --- | --- | --- | --- | --- | --- | --- | --- | --- |
| **H**^1^ |  |  | **Phenotype** | **BC** | **LW** | **A** | **H** | **H x A** |  |  |  |  |  |  |  |  |  |  |  |  |  |
|  |  |  |  |  |  |  |  |  |  |  |  |  |  |  |  | |  | |  | |  |
| h1 |  |  |  |  |  |  |  |  |  | 0 | 1 | 2 |  |  | 0 | | 1 | | 2 | |  |
|  |  |  | TDA | 0.333 | 0.043 | 0.084 | 0.961 | 0.001 |  | 70.0±3.32 | 64.5±7.72 | 73.0±21.78 |  | M | 57.2±4.39 | | 81.8±9.52 | | 65.2±30.69 | |  |
|  |  |  |  |  |  |  |  |  |  |  |  |  |  | Y | 82.7±6.44 | | 47.2±12.42 | | 80.9±31.35 | |  |
|  |  |  | P4CM | 0.070 | 0.245 | 0.114 | 0.852 | 0.007 |  | 0.81±0.01 | 0.82±0.02 | 0.79±0.08 |  | M | 0.85±0.01 | | 0.76±0.03 | | 0.87±0.11 | |  |
|  |  |  |  |  |  |  |  |  |  |  |  |  |  | Y | 0.77±0.02 | | 0.87±0.04 | | 0.71±0.11 | |  |
|  |  |  | OCM | 0.156 | 0.064 | 0.348 | 0.567 | 0.004 |  | 0.51±0.01 | 0.57±0.03 | 0.55±0.09 |  | M | 0.55±0.01 | | 0.49±0.04 | | 0.55±0.13 | |  |
|  |  |  |  |  |  |  |  |  |  |  |  |  |  | Y | 0.46±0.02**a** | | 0.66±0.05**b** | | 0.55±0.14**ab** | |  |
| h2 |  |  |  |  |  |  |  |  |  |  |  |  |  |  |  | |  | |  | |  |
|  |  |  | TDA | 0.431 | 0.014 | 0.110 | 0.205 | 0.838 |  | 67.0±3.36 | 75.8±8.15 | - |  | M | 60.0±4.35 | | 70.6±9.72 | | - | |  |
|  |  |  |  |  |  |  |  |  |  |  |  |  |  | Y | 73.9±6.48 | | 81.0±13.65 | | 135.8±44.46 | |  |
|  |  |  | P4CM | 0.107 | 0.124 | 0.137 | 0.363 | 0.655 |  | 0.81±0.01 | 0.81±0.03 | - |  | M | 0.84±0.01 | | 0.82±0.03 | | - | |  |
|  |  |  |  |  |  |  |  |  |  |  |  |  |  | Y | 0.79±0.02 | | 0.80±0.05 | | 0.55±0.16 | |  |
|  |  |  | OCM | 0.216 | 0.023 | 0.426 | 0.002 | 0.892 |  | 0.53±0.01**a** | 0.44±0.03**b** | - |  | M | 0.55±0.01 | | 0.45±0.04 | | - | |  |
|  |  |  |  |  |  |  |  |  |  |  |  |  |  | Y | 0.52±0.02 | | 0.43±0.06 | | 0.08±0.19 | |  |
| h3 |  |  |  |  |  |  |  |  |  |  |  |  |  |  |  | |  | |  | |  |
|  |  |  | TDA | 0.454 | 0.011 | 0.105 | 0.769 | 0.293 |  | 68.5±3.18 | 70.6±13.30 | - |  | M | 62.6±4.35 | | 50.5±13.99 | | - | |  |
|  |  |  |  |  |  |  |  |  |  |  |  |  |  | Y | 74.3±6.25 | | 90.6±22.52 | | - | |  |
|  |  |  | P4CM | 0.113 | 0.088 | 0.145 | 0.636 | 0.473 |  | 0.81±0.01 | 0.82±0.05 | - |  | M | 0.83±0.01 | | 0.88±0.05 | | - | |  |
|  |  |  |  |  |  |  |  |  |  |  |  |  |  | Y | 0.79±0.02 | | 0.76±0.08 | | - | |  |
|  |  |  | OCM | 0.236 | 0.043 | 0.290 | 0.401 | 0.767 |  | 0.52±0.01 | 0.47±0.06 | - |  | M | 0.54±0.01 | | 0.50±0.06 | | - | |  |
|  |  |  |  |  |  |  |  |  |  |  |  |  |  | Y | 0.50±0.02 | | 0.43±0.10 | | - | |  |
| h4 |  |  |  |  |  |  |  |  |  |  |  |  |  |  |  | |  | |  | |  |
|  |  |  | TDA | 0.435 | 0.010 | 0.114 | 0.393 | 0.130 |  | 67.2±3.28 | 73.5±10.70 | - |  | M | 62.2±4.21 | | 51.7±16.71 | | - | |  |
|  |  |  |  |  |  |  |  |  |  |  |  |  |  | Y | 72.1±6.45 | | 95.4±13.63 | | - | |  |
|  |  |  | P4CM | 0.116 | 0.084 | 0.131 | 0.793 | 0.089 |  | 0.82±0.01 | 0.82±0.04 | - |  | M | 0.83±0.01 | | 0.91±0.06 | | - | |  |
|  |  |  |  |  |  |  |  |  |  |  |  |  |  | Y | 0.80±0.02 | | 0.73±0.05 | | - | |  |
|  |  |  | OCM | 0.219 | 0.020 | 0.438 | 0.138 | 0.041 |  | 0.53±0.01 | 0.47±0.04 | - |  | M | 0.53±0.01 | | 0.58±0.07 | | - | |  |
|  |  |  |  |  |  |  |  |  |  |  |  |  |  | Y | 0.52±0.02 | | 0.37±0.06 | | - | |  |
| h5 |  |  |  |  |  |  |  |  |  |  |  |  |  |  |  | |  | |  | |  |
|  |  |  | TDA | 0.443 | 0.013 | 0.111 | 0.627 | - |  | 68.7±3.14 | - | - |  | M | 62.0±4.25 | | 54.2±15.66 | | - | |  |
|  |  |  |  |  |  |  |  |  |  |  |  |  |  | Y | 75.5±6.13 | | - | | - | |  |
|  |  |  | P4CM | 0.107 | 0.115 | 0.120 | 0.866 | - |  | 0.81±0.01 | - | - |  | M | 0.84±0.01 | | 0.83±0.05 | | - | |  |
|  |  |  |  |  |  |  |  |  |  |  |  |  |  | Y | 0.79±0.02 | | - | | - | |  |
|  |  |  | OCM | 0.233 | 0.028 | 0.394 | 0.586 | - |  | 0.52±0.01 | - | - |  | M | 0.53±0.01 | | 0.57±0.07 | | - | |  |
|  |  |  |  |  |  |  |  |  |  |  |  |  |  | Y | 0.50±0.02 | | - | | - | |  |
| h6 |  |  |  |  |  |  |  |  |  |  |  |  |  |  |  | |  | |  | |  |
|  |  |  | TDA | 0.460 | 0.014 | 0.093 | 0.685 | 0.984 |  | 68.2±3.24 | 72.6±10.81 | - |  | M | 61.2±4.25 | | 75.3±6.28 | | - | |  |
|  |  |  |  |  |  |  |  |  |  |  |  |  |  | Y | 65.8±13.57 | | 79.5±17.34 | | - | |  |
|  |  |  | P4CM | 0.126 | 0.105 | 0.124 | 0.465 | 0.733 |  | 0.81±0.01 | 0.79±0.04 | - |  | M | 0.84±0.01 | | 0.80±0.05 | | - | |  |
|  |  |  |  |  |  |  |  |  |  |  |  |  |  | Y | 0.79±0.02 | | 0.78±0.06 | | - | |  |
|  |  |  | OCM | 0.204 | 0.034 | 0.344 | 0.469 | 0.654 |  | 0.52±0.01 | 0.55±0.04 | - |  | M | 0.53±0.01 | | 0.59±0.06 | | - | |  |
|  |  |  |  |  |  |  |  |  |  |  |  |  |  | Y | 0.50±0.02 | | 0.51±0.07 | | - | |  |
| h7 |  |  |  |  |  |  |  |  |  |  |  |  |  |  |  | |  | |  | |  |
|  |  |  | TDA | 0.427 | 0.034 | 0.064 | 0.060 | 0.203 |  | 80.7±10.22 | 73.3±4.49 | 62.4±4.48 |  | M | 90.2±13.30 | | 64.1±5.31 | | 52.6±5.96 | |  |
|  |  |  |  |  |  |  |  |  |  |  |  |  |  | Y | 71.3±15.89 | | 82.5±7.97 | | 72.3±8.12 | |  |
|  |  |  | P4CM | 0.104 | 0.180 | 0.093 | 0.150 | 0.093 |  | 0.78±0.03 | 0.80±0.01 | 0.83±0.01 |  | M | 0.72±0.05 | | 0.83±0.02 | | 0.86±0.02 | |  |
|  |  |  |  |  |  |  |  |  |  |  |  |  |  | Y | 0.83±0.06 | | 0.76±0.03 | | 0.80±0.03 | |  |
|  |  |  | OCM | 0.239 | 0.068 | 0.252 | 0.015 | 0.923 |  | 0.44±0.04 | 0.49±0.02 | 0.55±0.02 |  | M | 0.44±0.06 | | 0.52±0.02 | | 0.58±0.02 | |  |
| h8 |  |  |  |  |  |  |  |  |  |  |  |  |  | Y | 0.43±0.07 | | 0.47±0.03 | | 0.53±0.03 | |  |
|  |  |  | TDA | 0.411 | 0.012 | 0.063 | 0.061 | 0.038 |  | 68±3.1 | 68.6±24.1 | - |  | M | 59.9±4.13 | | 110.8±19.57 | | - | |  |
|  |  |  |  |  |  |  |  |  |  |  |  |  |  | Y | 76.1±6.04 | | 26.5±44.23 | | - | |  |
|  |  |  | P4CM | 0.103 | 0.090 | 0.095 | 0.182 | 0.016 |  | 0.81±0.01 | 0.87±0.09 | - |  | M | 0.84±0.01 | | 0.68±0.07 | | - | |  |
|  |  |  |  |  |  |  |  |  |  |  |  |  |  | Y | 0.79±0.02 | | 1.06±0.16 | | - | |  |
|  |  |  | OCM | 0.181 | 0.047 | 0.233 | 0.004 | 0.751 |  | 0.52±0.01**a** | 0.26±0.10**b** | - |  | M | 0.55±0.01 | | 0.32±0.08 | | - | |  |
|  |  |  |  |  |  |  |  |  |  |  |  |  |  | Y | 0.50±0.02 | | 0.21±0.19 | | - | |  |
|  |  |  |  |  |  |  |  |  |  |  |  |  |  |  |  |  | |  | |  | |

^1^Block 0: snp_ex4 - snp_ex7 -snp_ex8 - snp_ex20_1 - snp_ex20_2 - snp_ex20_3.

^2^ 0 copy: LSMs and SE for 0 copy of the haplotype; 1 copy: LSMs and SE for 1 copy of the haplotype; and 2 copies: LSMs and SE for 2 copies of the haplotype

^3^M=mature; Y=young
