# Supplementary material for: The LEPR Gene Is Associated with Reproductive Seasonality Traits in Rasa Aragonesa Sheep
Source: Animals (Basel). 2020 Dec 21;10(12):2448. doi: 10.3390/ani10122448 (PMC7766475; doi:10.3390/ani10122448)
Supplement: Supplementary file 1 [file animals-10-02448-s001.zip › Table S5.docx]

**Table S5.** Type III test for the body condition (BC), live weight (LW), age (A), haplotype (H), and haplotype x age (H x A) effects for the Block 1 haplotype using the seasonality phenotype data from Rasa Aragonesa ewes. The least square means and standard errors of the *LEPR* polymorphisms in the seasonality phenotype data in Rasa Aragonesa ewes are also shown.

|  |  |  | **P value** | | | | | |  |  | **LSMs H**^2^ | | |  | **A**^3^ | **LSMs H x A**^2^ | | |
| --- | --- | --- | --- | --- | --- | --- | --- | --- | --- | --- | --- | --- | --- | --- | --- | --- | --- | --- |
| **H**^1^ |  |  | **Phenotype** | **BC** | **LW** | **A** | **H** | **H x A** |  |  |  |  |  |  |  |  |  |  |
|  |  |  |  |  |  |  |  |  |  |  |  |  |  |  |  |  |  |  |
| h1 |  |  |  |  |  |  |  |  |  |  | 0 | 1 | 2 |  |  | 0 | 1 | 2 |
|  |  |  | TDA | 0.374 | 0.015 | 0.121 | 0.072 | 0.644 |  |  | 66.3±3.34 | 82.4±8.22 | - |  | M | 60.4±4.28 | 72.4±11.53 | - |
|  |  |  |  |  |  |  |  |  |  |  |  |  |  |  | Y | 72.2±6.49 | 92.4±12.28 | - |
|  |  |  | P4CM | 0.098 | 0.112 | 0.140 | 0.381 | 0.639 |  |  | 0.82±0.01 | 0.79±0.03 | - |  | M | 0.84±0.01 | 0.82±0.04 | - |
|  |  |  |  |  |  |  |  |  |  |  |  |  |  |  | Y | 0.80±0.02 | 0.75±0.04 | - |
|  |  |  | OCM | 0.162 | 0.028 | 0.460 | 0.003 | 0.078 |  |  | 0.55±0.01**a** | 0.42±0.03**b** | - |  | M | 0.54±0.01 | 0.49±0.05 | - |
|  |  |  |  |  |  |  |  |  |  |  |  |  |  |  | Y | 0.53±0.02 | 0.34±0.05 | - |
| h2 |  |  |  |  |  |  |  |  |  |  |  |  |  |  |  |  |  |  |
|  |  |  | TDA | 0.425 | 0.015 | 0.081 | 0.559 | - |  |  | 68.3±3.15 | - | - |  | M | 60.9±4.25 | 69.4±14.08 | - |
|  |  |  |  |  |  |  |  |  |  |  |  |  |  |  | Y | 75.7±6.12 | - | - |
|  |  |  | P4CM | 0.101 | 0.118 | 0.092 | 0.241 | - |  |  | 0.81±0.01 | - | - |  | M | 0.84±0.01 | 0.78±0.05 | - |
|  |  |  |  |  |  |  |  |  |  |  |  |  |  |  | Y | 0.79±0.02 | - | - |
|  |  |  | OCM | 0.224 | 0.032 | 0.332 | 0.793 | - |  |  | 0.52±0.01 | - | - |  | M | 0.54±0.01 | 0.52±0.06 | - |
|  |  |  |  |  |  |  |  |  |  |  |  |  |  |  | Y | 0.50±0.02 | - | - |
| h3 |  |  |  |  |  |  |  |  |  |  |  |  |  |  |  |  |  |  |
|  |  |  | TDA | 0.442 | 0.016 | 0.086 | 0.763 | 0.868 |  |  | 68.3±3.25 | 70.6±10.66 | - |  | M | 61.2±4.29 | 61.7±13.01 | 93.1±44.36 |
|  |  |  |  |  |  |  |  |  |  |  |  |  |  |  | Y | 75.4±6.29 | 79.6±17.36 | - |
|  |  |  | P4CM | 0.124 | 0.112 | 0.115 | 0.660 | 0.772 |  |  | 0.81±0.01 | 0.79±0.04 | - |  | M | 0.84±0.01 | 0.80±0.04 | 0.74±0.16 |
|  |  |  |  |  |  |  |  |  |  |  |  |  |  |  | Y | 0.79±0.02 | 0.78±0.06 | - |
|  |  |  | OCM | 0.202 | 0.035 | 0.342 | 0.711 | 0.636 |  |  | 0.52±0.01 | 0.55±0.04 | - |  | M | 0.53±0.01 | 0.59±0.05 | 0.49±0.20 |
|  |  |  |  |  |  |  |  |  |  |  |  |  |  |  | Y | 0.50±0.02 | 0.51±0.07 | - |
| h4 |  |  |  |  |  |  |  |  |  |  |  |  |  |  |  |  |  |  |
|  |  |  | TDA | 0.448 | 0.015 | 0.082 | 0.178 | 0.700 |  |  | 103.7±25.47 | 76.2±6.42 | 65.7±3.52 |  | M | 75.1±25.57 | 68.8±7.76 | 58.9±4.63 |
|  |  |  |  |  |  |  |  |  |  |  |  |  |  |  | Y | 132.2±44.19 | 83.5±10.88 | 72.5±6.66 |
|  |  |  | P4CM | 0.109 | 0.122 | 0.109 | 0.220 | 0.776 |  |  | 0.73±0.09 | 0.78±0.02 | 0.82±0.01 |  | M | 0.80±0.09 | 0.80±0.02 | 0.85±0.01 |
|  |  |  |  |  |  |  |  |  |  |  |  |  |  |  | Y | 0.65±0.16 | 0.77±0.04 | 0.79±0.02 |
|  |  |  | OCM | 0.223 | 0.035 | 0.309 | 0.161 | 0.171 |  |  | 0.48±0.11 | 0.46±0.02 | 0.54±0.01 |  | M | 0.55±0.11 | 0.52±0.03 | 0.54±0.02 |
|  |  |  |  |  |  |  |  |  |  |  |  |  |  |  | Y | 0.41±0.19 | 0.40±0.04 | 0.53±0.02 |

^1^Block 1: snp_ex4 - snp_ex7 -snp_ex8.

^2^ 0 copy: LSMs and SE for 0 copy of the haplotype; 1 copy: LSMs and SE for 1 copy of the haplotype; and 2 copies: LSMs and SE for 2 copies of the haplotype

^3^M=mature; Y=young
