# Supplementary material for: The LEPR Gene Is Associated with Reproductive Seasonality Traits in Rasa Aragonesa Sheep
Source: Animals (Basel). 2020 Dec 21;10(12):2448. doi: 10.3390/ani10122448 (PMC7766475; doi:10.3390/ani10122448)
Supplement: Supplementary file 1 [file animals-10-02448-s001.zip › Table S6.docx]

**Table S6.** Type III test for the body condition (BC), live weight (LW), age (A), haplotype (H), and haplotype x age (H x A) effects for the Block 2 haplotype using the seasonality phenotype data from Rasa Aragonesa ewes. The least square means and standard errors of the *LEPR* polymorphisms in the seasonality phenotype data in Rasa Aragonesa ewes are also shown.

| **H**^1^ |  |  | **P value** | | | | | |  | **LSMs H**^2^ | | | |  | **A**^3^ | **LSMs H x A**^2^ | | |
| --- | --- | --- | --- | --- | --- | --- | --- | --- | --- | --- | --- | --- | --- | --- | --- | --- | --- | --- |
|  |  |  | **Phenotype** | **BC** | **LW** | **A** | **H** | **H x A** |  |  |  |  |  |  |  |  |  |  |
|  |  |  |  |  |  |  |  |  |  |  |  |  |  |  |  |  |  |  |
| h1 |  |  |  |  |  |  |  |  |  |  | 0 | 1 | 2 |  |  | 0 | 1 | 2 |
|  |  |  | TDA | 0.336 | 0.049 | 0.064 | 0.597 | 0.0003 |  |  | 69.8±3.33 | 64.3±7.24 | 84.7±19.82 |  | M | 55.6±4.46 | 82.2±8.69 | 88.0±24.79 |
|  |  |  |  |  |  |  |  |  |  |  |  |  |  |  | Y | 83.9±6.38**a** | 46.3±11.97**b** | 81.4±31.09**ab** |
|  |  |  | P4CM | 0.070 | 0.254 | 0.094 | 0.583 | 0.001 |  |  | 0.81±0.01 | 0.82±0.02 | 0.75±0.07 |  | M | 0.86±0.01 | 0.76±0.03 | 0.78±0.09 |
|  |  |  |  |  |  |  |  |  |  |  |  |  |  |  | Y | 0.76±0.02 | 0.88±0.04 | 0.71±0.11 |
|  |  |  | OCM | 0.197 | 0.048 | 0.335 | 0.667 | 0.010 |  |  | 0.51±0.01 | 0.56±0.03 | 0.48±0.09 |  | M | 0.55±0.02 | 0.50±0.04 | 0.40±0.11 |
|  |  |  |  |  |  |  |  |  |  |  |  |  |  |  | Y | 0.47±0.02 | 0.62±0.05 | 0.56±0.14 |
| h2 |  |  |  |  |  |  |  |  |  |  |  |  |  |  |  |  |  |  |
|  |  |  | TDA | 0.454 | 0.018 | 0.099 | 0.207 | 0.840 |  |  | 67.2±3.38 | 76±8.16 | - |  | M | 60±4.39 | 70.6±9.75 | - |
|  |  |  |  |  |  |  |  |  |  |  |  |  |  |  | Y | 74.4±6.48 | 81.4±13.67 | 136.4±44.54 |
|  |  |  | P4CM | 0.115 | 0.134 | 0.130 | 0.363 | 0.654 |  |  | 0.81±0.01 | 0.81±0.03 | - |  | M | 0.84±0.01 | 0.82±0.03 | - |
|  |  |  |  |  |  |  |  |  |  |  |  |  |  |  | Y | 0.79±0.02 | 0.80±0.05 | 0.55±0.16 |
|  |  |  | OCM | 0.239 | 0.020 | 0.432 | 0.002 | 0.876 |  |  | 0.54±0.01**a** | 0.44±0.03**b** | - |  | M | 0.84±0.01 | 0.82±0.03 | - |
|  |  |  |  |  |  |  |  |  |  |  |  |  |  |  | Y | 0.79±0.02 | 0.80±0.05 | 0.55±0.16 |
| h3 |  |  |  |  |  |  |  |  |  |  |  |  |  |  |  |  |  |  |
|  |  |  | TDA | 0.464 | 0.019 | 0.075 | 0.596 | 0.275 |  |  | 68.1±3.22 | 78.2±11.08 | - |  | M | 61.7±4.40 | 59.3±12.29 | - |
|  |  |  |  |  |  |  |  |  |  |  |  |  |  |  | Y | 74.5±6.27 | 97±18.55 | - |
|  |  |  | P4CM | 0.119 | 0.125 | 0.111 | 0.744 | 0.576 |  |  | 0.81±0.01 | 0.79±0.04 | - |  | M | 0.84±0.01 | 0.84±0.04 | - |
|  |  |  |  |  |  |  |  |  |  |  |  |  |  |  | Y | 0.79±0.02 | 0.74±0.07 | - |
|  |  |  | OCM | 0.265 | 0.039 | 0.264 | 0.089 | 0.552 |  |  | 0.53±0.01 | 0.43±0.04 | - |  | M | 0.54±0.01 | 0.48±0.05 | - |
|  |  |  |  |  |  |  |  |  |  |  |  |  |  |  | Y | 0.51±0.02 | 0.38±0.08 | - |
| h4 |  |  |  |  |  |  |  |  |  |  |  |  |  |  |  |  |  |  |
|  |  |  | TDA | 0.406 | 0.044 | 0.060 | 0.085 | 0.092 |  |  | 92.6±14.20 | 69.8±5.13 | 67.0±3.88 |  | M | 103.4±17.86 | 67.5±6.17 | 53.9±5.18 |
|  |  |  |  |  |  |  |  |  |  |  |  |  |  |  | Y | 81.8±22.32 | 72.1±8.90 | 80.1±7.18 |
|  |  |  | P4CM | 0.091 | 0.215 | 0.094 | 0.154 | 0.139 |  |  | 0.72±0.05 | 0.82±0.01 | 0.81±0.01 |  | M | 0.70±0.06 | 0.82±0.02 | 0.86±0.01 |
|  |  |  |  |  |  |  |  |  |  |  |  |  |  |  | Y | 0.74±0.08 | 0.81±0.03 | 0.77±0.02 |
|  |  |  | OCM | 0.218 | 0.067 | 0.252 | 0.021 | 0.218 |  |  | 0.42±0.06 | 0.49±0.02 | 0.54±0.01 |  | M | 0.38±0.08 | 0.49±0.02 | 0.58±0.02 |
|  |  |  |  |  |  |  |  |  |  |  |  |  |  |  | Y | 0.45±0.10 | 0.49±0.04 | 0.50±0.03 |

^1^ Block2: snp_ex20_1 -snp_ex20_2 - snp_ex20_3.

^2^ 0 copy: LSMs and SE for 0 copy of the haplotype; 1 copy: LSMs and SE for 1 copy of the haplotype; and 2 copies: LSMs and SE for 2 copies of the haplotype

^3^M=mature; Y=young
